# The Well-Being Coaching Inventory (WCI): Questionnaire Development and Validation

**DOI:** 10.1177/15598276251320573

**Published:** 2025-02-20

**Authors:** Sebastian Harenberg, Gary Sforzo, Rosie Hunter, Erika Jackson, Margaret Moore

**Affiliations:** 1 1270 Department of Human Kinetics, St. Francis Xavier University, Antigonish, NS, Canada (SH); 2 4102 Department of Exercise Science and Athletic Training, Ithaca College, Ithaca, NY, USA (GS); 3 Wellcoaches Corporation, Wellesley, MA, USA (RH, EJ, MM)

**Keywords:** health and well-being coaching, employee well-being, work-life, mind-body, validation study, core outcome measure, coaching practice

## Abstract

**Objective:**

The aim of the present study was to psychometrically test and validate the Well-being Coaching Inventory (WCI), a proposed measure of interconnected, whole-person well-being in the context of health and wellness coaching (HWC).

**Methods:**

Initially 49 items, the WCI was conceived with 4 dimensions: Mind, Body, Work, and Life. The inventory was evaluated in 3 sequential studies to test: (a) face validity, (b) convergent validity, and (c) predictive validity. Expert judgment, correlational analyses, and factor analyses were techniques applied to collected WCI data.

**Results:**

After statistical evaluation (n = 261) of fit to each dimension, the WCI was shortened to 20 items that demonstrated convergent validity. Further use of confirmatory factor analyses and exploratory structural equation model in a large sample study (n = 531) provided additional support for the inventory’s convergent validity. Through correlation analyses to theoretically related concepts predictive validity was established.

**Conclusions:**

The WCI is a valid, applicable, and reliable scale for use in HWC research and practice. It is an instrument that will aid HWC practitioners and researchers as a central outcome measure for their practice.

“The WCI query of love, hope, meaning, gratitude, and compassion is considered to embody an expression of spirituality.”

## Introduction

Health and well-being coaching (HWC) is an increasingly adopted health care intervention demonstrating beneficial results in a variety of patient populations. The National Board for Health and Wellness Coaching (NBHWC) defines the role of the coach as one who “supports clients in activating internal strengths and external resources to make sustainable and healthy lifestyle behavior changes.”^1^ Since 2017, NBHWC has certified over 10,000 HWC professionals.^2^ In 2019, the American Medical Association (AMA) approved current procedural terminology (CPT) codes for HWC services. Moreover, in 2024 HWC services were added to the Medicare Telehealth Services list by the Centers for Medicare and Medicaid Services (CMS).

Examining collected HWC research,^3^ HWC is effective at improving a wide diversity of health outcomes. These are usually patient-related, with body weight, blood pressure, and hemoglobin A1C measurements typically cited for obese, hypertensive, and diabetic patients, respectively. However, the coaching process impacts more than biometrics, potentially extending to psychological variables and many aspects of work-life. A good example of far-reaching coaching effects is seen in studies of clinician burnout.^4-6^ McGonagle et al. showed in 50 primary care physicians that 6 coaching sessions over 3 months significantly improved burnout scores, work engagement and psychological capital (i.e., resilience, hope, optimism, but not efficacy).^4^ The authors recommended organizations make HWC available to promote clinician well-being. Outcomes from this study well-defined clinician burnout and HWC effects on important work-related functions. However, the beneficial effects of coaching on other critical aspects of work and life which impact health and performance, beyond burnout and psychological capital, were not as easily captured.

There is a growing interest in a whole-person approach to health and well-being that includes mind and body functioning at work as well as in personal life.^7^ HWC impacts whole-person health and well-being but to date, there are no tools developed to measure these potential wide-reaching coaching effects. Specifically, an instrument is needed to target health and performance impact variables, that improve internal strengths and external resources of the whole client or patient, connected to both work and personal life.

**Figure 1.**
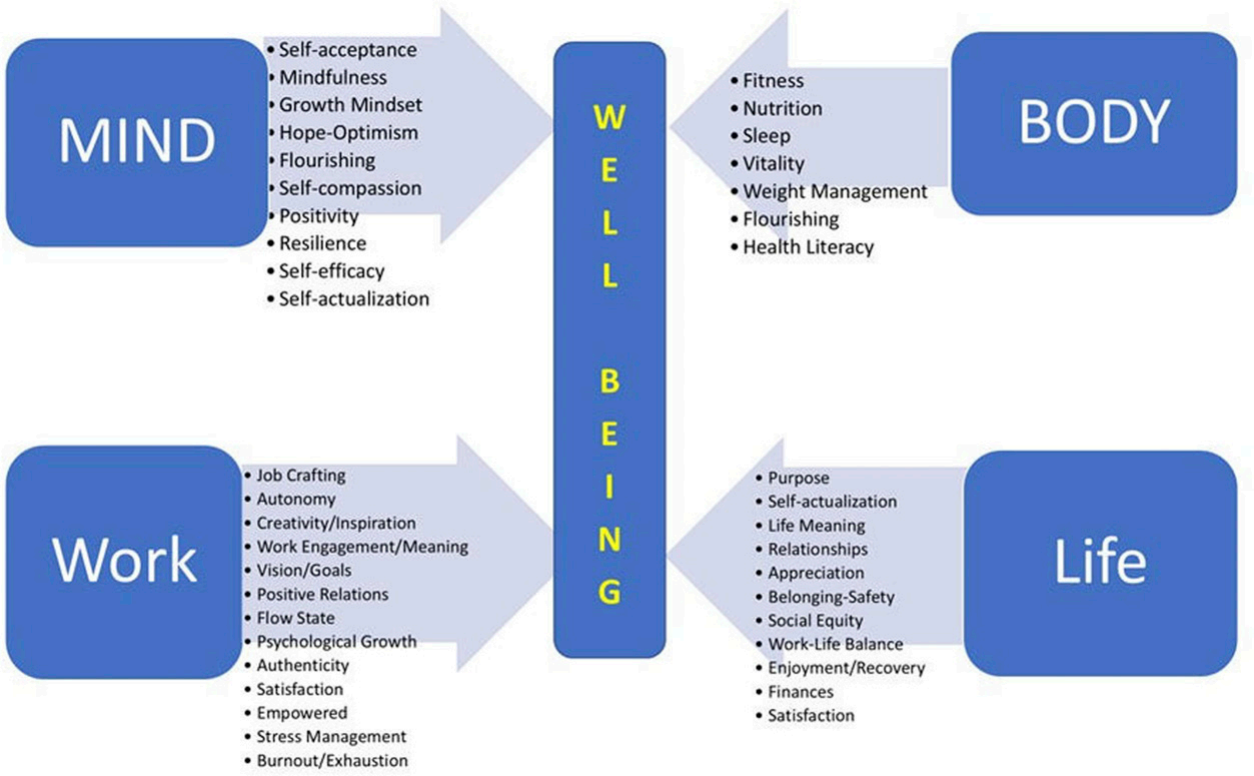
WCI Subdimensions and constructs.

The Well-being Coaching Inventory (WCI) was created to support coaching an interconnected whole person while addressing critical determinants of well-being that affect health and performance. The intention was to build an HWC-related assessment, distinct from other health risk or healthy lifestyle assessments. Leveraging a team of HWC experts and more than 30 recognized well-being measures, the WCI was developed as a set of 49 questions. The inventory is made up of 4 dimensions of personal health and well-being (Mind, Body, Work, Life) querying 47 well-established constructs. Note, the term “life” is not meant to subsume the totality of life but instead the “life” subdimension refers to one’s personal life, outside work or school. The complete structure of the WCI is illustrated in Figure 1. The WCI explores issues related to psychosocial theories, such as emotional and social intelligence, self-determination, mindfulness, and self-efficacy. Individually, these constructs are well-researched and most have an independent and validated measurement process. Collectively, they form an original group of HWC-related concepts highly connected to work well-being and overall well-being. The 47 psychological constructs addressed by the WCI are keys to one’s thriving and flourishing. As such, the WCI allows a much wider range of coaching benefits to be inspected than other questionnaires not specifically designed for HWC. Greater detail on how the WCI was created can be found in the Methods section.

The purpose of the present study was to test the 49 WCI items psychometrically and to establish various forms of instrument validity. If validated, the WCI can provide a psychometrically sound outcome measure, which captures coaching-specific effects more fully than any single existing measure. The WCI would serve to complement patient-specific outcomes (e.g., BP in hypertension) as a central measure in HWC research while also providing a useful tool for application by coaching professionals.

## Methods

### Well-Being Coaching Inventory (WCI)

The WCI was created by leveraging a core team (MM, EJ, RH) of HWC experts with more than 50 years of collective experience in HWC research, research translation, education, and clinical application. Before deciding to build a HWC inventory, the team conducted an extensive review of over 30 existing surveys with relevance to HWC and well-being. These surveys represented a diversity of approaches: government-developed or financed surveys (from countries including the United States, Canada, and Denmark), surveys developed by academics, and surveys developed by organizations. Some surveys addressed a particular construct (e.g., burnout, positivity, resilience, self-actualization, flourishing, job crafting) while others more broadly assessed health and well-being. None of these questionnaires, however, were specifically designed to assess HWC, as is the case when these constructs are brought together in the WCI.

Next, a group of experienced coaches and researchers were recruited to review the surveys collected and provide feedback on relevance to HWC. Considering this input, the core team concluded that no single existing survey fit HWC needs, and none were specifically designed for HWC interventions. It was determined that a new instrument with specific relevance to HWC needed to be developed, while drawing on the plethora of existing validated assessments. The core team initially developed a set of 49 questions with items expected to be highly relevant to HWC processes. This original version of the WCI can be accessed at https://survey.alchemer.com/s3/7971712/Wellcoaches-Well-being-Inventory.

The steps undertaken to test the psychometric features and validate the WCI in 3 sequential studies are described below. The overall strategy throughout the process was guided by recommendations from the psychometric literature.^8,9^ The STROBE guidelines for observational studies were used for the outlined projects. All studies were reviewed and approved by the Institutional Review Board at St. Francis Xavier University and all participants provided informed consent.

Three types of validity were investigated: (a) Face validity, or the measure’s ability to capture the true essence of the construct under examination. Expert judgments and ratings are commonly used to assess this type of validity,^10^ which was done in Study 1. (b) Convergent validity, which is concerned with the selection of items that best represent an unobservable (perceived) variable. Correlation analysis and factor analytical techniques (e.g., confirmatory factor analysis, exploratory structural equation modeling), which were used in Studies 2 and 3, are the preferred choice to establish convergent validity.^10^ (c) Predictive validity, or the degree to which the created scale is associated with other measures of different constructs, according to theory. These associations are commonly tested via correlation analysis, which is outlined in Study 3.^10^

### Study 1

#### Objective

The primary objective of Study 1 was to establish face validity of the 49 developed items of the WCI.

#### Participants and Procedure

To obtain the perspective of experts, email invitations were sent to experienced, credentialed coaches: 1. Health and well-being coaches trained and certified by Wellcoaches Corporation, a coaching school of health professionals. 2. International Coach Federation-certified coaches contracted by AceUp, a leadership coaching organization. In total, 52 (female n = 38; male n = 13, non-binary = 1, Did not report = 1) coaches viewed an online version of the WCI via Qualtrics.

On average the participants were 56.18 ± 7.43 years old. The majority of the responding participants were white (n = 41), followed by black (n = 3), Asian (n = 2), and other ethnicity (n = 3). The majority held a Master’s (n = 25) or doctoral degree (n = 18), as the highest degree earned, followed by a bachelor’s (n = 7) or a high-school degree (n = 2). Fifty participants practiced in the United States, spanning over 21 states. One coach practiced in Canada and one in Australia. On average, the participants had 11.65 ± 6.64 years of HWC experience.

#### Measures

The WCI consisted of 49 statements (e.g., “I handle setbacks as learning opportunities.”), comprising physical, mental, work, and life subscales. The participants were presented with each of the 49 statements and asked to rate them on single-item measures reflecting applicability (i.e., “This statement is applicable”), clarity (i.e., “This statement is clear”), and readability (i.e., “This statement is readable”). Each of the items was measured with a Likert scale, ranging from 1—Strongly disagree to 5—Strongly agree. After each statement, participants were invited to provide comments as to how the statement could be improved. At the end of the survey, an open-ended box for comments or suggestions for further inclusions was provided.

#### Statistical Analysis

Descriptive analysis (i.e., calculation of means and standard deviations) was conducted for each item (i.e., applicability, clarity, readability). In addition, the number of participants rating a particular item at 3 (neutral point on the Likert scale) or below was counted. Qualitative comments on rewording or addition of items were analyzed by frequency and content applicability.

#### Results & Discussion

The participants rated all items generally as applicable, clear, and readable. The averaged ratings ranged from 3.96 – 5.00, corresponding with responses ranging from “agree” to “strongly agree.” Rather than relying solely on averaged responses, ratings of neutral (i.e., 3/5) or below were counted. These responses for the individual scores on applicability, clarity, and readability ranged from 0 to 17. The average scores and counts can be found in Table 1. About half of participants (n = 25) also shared many qualitative suggestions and comments on how the items could be improved.

**Table 1.** Expert Ratings.

	Applicability		Clarity		Readability	
	Mean ± SD	Number of Ratings ≦ 3	Mean ± SD	Number of Ratings ≦ 3	Mean ± SD	Number of Ratings ≦ 3
Item 1	4.83 ± .51	3	4.89 ± .38	1	4.92 ± .27	0
Item 2	4.71 ± .61	1	4.71 ± .64	3	4.87 ± .35	0
Item 3	4.77 ± . 51	2	4.64 ± .74	4	4.85 ± .36	0
Item 4	4.83 ± . 47	2	4.83 ± .47	2	4.81 ± .49	2
Item 5	4.88 ± . 48	1	4.77 ± .59	2	4.86 ± .45	2
Item 6	4.73 ± . 63	3	4.35 ± 1.01	9	4.69 ± .70	3
Item 7	4.66 ± . 72	5	4.30 ± 1.07	11	4.60 ± .73	5
Item 8	4.58 ± . 80	3	4.25 ± 1.01	10	4.61 ± .70	4
Item 9	4.86 ± .40	1	4.90 ± .36	1	5	0
Item 10	4.46 ± .87	7	3.96 ± 1.24	15	4.31 ± 1.10	11
Item 11	4.92 ± .27	0	4.85 ± .50	1	4.87 ± .53	2
Item 12	4.83 ± .51	3	4.89 ± .38	1	4.92 ± .27	0
Item 13	4.62 ± .95	6	4.73 ± .70	3	4.94 ± .24	0
Item 14	4.57 ± .76	6	4.48 ± .96	6	4.69 ± .70	1
Item 15	4.49 ± 1.08	7	4.52 ± .96	6	4.61 ± .95	6
Item 16	4.57 ± .86	5	4.56 ± 1.00	6	4.77 ± .61	3
Item 17	4.60 ± .96	6	4.65 ± .84	3	4.83 ± .62	2
Item 18	4.84 ± .51	1	4.86 ± .53	2	4.85 ± .64	2
Item 19	4.80 ± .54	3	4.52 ± 1.13	7	4.63 ± 1.00	5
Item 20	4.71 ± .78	5	4.67 ± .84	5	4.86 ± .45	2
Item 21	4.78 ± .51	2	4.66 ± .85	5	4.82 ± .69	4
Item 22	4.60 ± .80	6	3.96 ± 1.40	17	4.33 ± 1.08	11
Item 23	4.62 ± .75	3	4.46 ± .90	8	4.60 ± .85	6
Item 24	4.55 ± .76	4	4.16 ± 1.17	12	4.39 ± 1.04	8
Item 25	4.81 ± .53	3	4.77 ± .68	2	4.83 ± .62	1
Item 26	4.85 ± .46	2	4.84 ± .51	1	4.89 ± .47	1
Item 27	4.66 ± .66	3	4.65 ± .89	3	4.74 ± .73	3
Item 28	4.82 ± .43	1	4.75 ± .74	2	4.77 ± .74	2
Item 29	4.86 ± .45	2	4.71 ± .83	3	4.78 ± .76	3
Item 30	4.96 ± .20	0	4.90 ± .46	1	4.92 ± .44	1
Item 31	4.75 ± .60	4	4.71 ± .86	4	4.77 ± .76	3
Item 32	4.27 ± 1.07	10	4.16 ± 1.22	11	4.49 ± 1.05	7
Item 33	4.75 ± .63	3	4.69 ± .84	5	4.78 ± .70	3
Item 34	4.78 ± .58	2	4.73 ± .70	3	4.82 ± .56	2
Item 35	4.72 ± .78	3	4.61 ± .92	4	4.73 ± .83	3
Item 36	4.74 ± .24	0	4.94 ± .24	0	4.96 ± .19	0
Item 37	4.67 ± .86	3	4.65 ± .88	5	4.75 ± .79	4
Item 38	4.83 ± .65	2	4.89 ± .32	0	4.94 ± .24	0
Item 39	4.87 ± .40	1	4.75 ± .74	3	4.83 ± .62	2
Item 40	4.73 ± .57	3	4.42 ± 1.09	7	4.82 ± .43	1
Item 41	4.77 ± .61	3	4.87 ± .40	1	4.92 ± .33	1
Item 42	4.78 ± .70	3	4.50 ± 1.06	7	4.71 ± .81	4
Item 43	4.73 ± .78	3	4.56 ± 1.02	6	4.67 ± .92	4
Item 44	4.82 ± .62	1	4.55 ± 1.01	7	4.71 ± .81	4
Item 45	4.73 ± .78	3	4.39 ± 1.12	9	4.59 ± 1.00	5
Item 46	4.63 ± .91	5	4.33 ± 1.25	10	4.46 ± 1.16	9
Item 47	4.80 ± .53	1	4.67 ± .79	4	4.77 ± .74	3
Item 48	4.57 ± .90	5	4.45 ± .92	7	4.59 ± .83	5
Item 49	4.92 ± .34	1	4.92 ± .27	1	4.96 ± .20	0

Based on the average responses, counts of neutral responses or below, as well as qualitative comments, the research team engaged in a discussion that included a re-evaluation of the purpose and wording of each item, as well as possible modifications. As a result, 5 items remained the same and 44 items were revised. The modifications included changes in wording (e.g., resolving double-barreled questions, clearer choices of words) and/or simplifications (e.g., reducing the number of words). The goal was to arrive at items that reflect the intended purpose in the most understandable and clear way possible.^9^ One item (i.e., I am fully present, attentive, and focused on the activity in which I am engaged in the moment.) was split into 2 items (i.e., sense of presence, engagement in activities) as it reflected 2 separate constructs. Based on the comments from participants, an item was added (“I spend time in nature regularly”). Lastly 2 items were removed (“I feel safe in my home and in my community.”; “I feel a sense of belonging at home and in my community”) because they reflected constructs outside of the scope of outcomes for HWC. All items and modifications can be found in Table 2.

**Table 2.** Items and Modifications by Studies.

Dimension	Study 1 - Phase 1 (49 item)	Modification	Study 2 - Phase 2&3 (49 item)	Modification	Study 2 & 3 (20 Item)
Mental	1. I am fully present, attentive, and focused on the activity in which I am engaged in the moment	Split into 1. and 2	1. My mind is present throughout the day		1. My mind is present throughout the day
			2. I am actively engaged in my activities throughout the day	High modification index	
Mental	2. I accept myself, including all of my quirks and limitations	Revised: Wording	3. I accept myself as I am, with my strengths and limitations		2. I accept myself as I am, with my strengths and limitations
Mental	3. When faced with adversity and roadblocks, I feel confident that I can handle them	Revised: Simplified	4. I feel confident that I can overcome adversity	High modification index	
Mental	4. I handle setbacks as learning opportunities	Revised: Wording	5. I view setbacks as learning opportunities		3. I view setbacks as learning opportunities
Mental	5. I am aware of my triggers for stress	Revised: Simplified	6. I am aware of what increases my stress	Item-total correlation low	
Mental	6. I use healthy ways to minimize stress	Revised: Reworded	7. I can calm myself down when I feel stressed or anxious		4. I can calm myself down when I feel stressed or anxious
Mental	7. I generally avoid addictive substances and behaviors	Revised: Expanded	8. I notice when I feel addicted to something (e.g., work, substances, social media)	High modification index	
Mental	8. I feel kind and compassionate towards my own suffering	Revised: Simplified	9. I am kind to myself in difficult times		5. I am kind to myself in difficult times
Mental	9. I feel confident that I can accomplish what I set out to do	Kept the same	10. I feel confident that I can accomplish what I set out to do	Low factor loading	
Mental	10. I cultivate positive feeling related to my past	Revised: Simplified	11. My feelings about my past are mostly positive	High modification index	
Mental	11. I savor small pleasures in life	Revised: Wording	12. I enjoy the small pleasures in life	High modification index	
Mental	12. I feel hopeful and optimistic about my future	Revised: Wording	13. I feel hopeful about my future	High modification index	
Mental	13. Overall, my mental health is good	Revised: Wording	14. Overall, I feel mentally healthy	High modification index	
Physical	14. I feel good energy and vitality all day	Revised: Simplified	15. I feel energetic most days	Low factor loading	
Physical	15. I exercise my body vigorously at least 3 days a week	Revised: Simplified	16. I am physically active most days		6. I am physically active most days
Physical	16. I eat plenty of plant-based foods—vegetables, fruits, beans and lentils, whole grains, nuts and seeds	Revised: Simplified	17. I eat several servings of vegetables/fruits daily		7. I eat several servings of vegetables/fruits daily
Physical	17. I maintain a healthy weight	Revised: Wording	18. I feel I maintain a healthy weight		8. I feel I maintain a healthy weight
Physical	18. I get a healthy amount of sleep (7-9 hours) most nights	Revised: Simplified	19. I feel rested after a night’s sleep	High modification index	
Physical	19. I take good care of my physical health: getting tests, seeking medical advice and managing health issues promptly	Revised: Simplified	20. I seek medical care promptly for health issues	Item-total correlation low	
Physical		Added based on comments	21. I spend time in nature regularly		9. I spend time in nature regularly
Physical	20. Overall, my physical health is good	Revised: Wording	22. Overall, I feel physically healthy	High modification index	
Work	21. I have clear professional and career goals	Revised: Wording	23. I have clear goals for my work-life	High modification index	
Work	22. I have the inspiration to be energized at work	Revised: Wording	24. I feel inspired to be my best at work		10. I feel inspired to be my best at work
Work	23. I have enough freedom to make my own choices about my duties at work	Revised: Wording	25. I have enough freedom to choose how I do my job		11. I have enough freedom to choose how I do my job
Work	24. I design my work to be fulfilling	Revised: Wording	26. I find ways to make my work fulfilling		12. I find ways to make my work fulfilling
Work	25. I feel empowered at work	Revised: Wording	27. I feel empowered to do my best at work	High inter-item correlation	
Work	26. I am satisfied with my work performance	Kept the same	28. I am satisfied with my work performance		13. I am satisfied with my work performance
Work	27. At work, I honestly and freely share my values, perspectives and what is important to me	Revised: Simplified	29. At work, I am able to share what is important to me		14. At work, I am able to share what is important to me
Work	28. I have good relationships at work—positive, supportive, collaborative	Revised: Wording	30. I have positive relationships at work	High modification index	
Work	29. I am engaged at work, using my skills and strengths	Revised: Wording	31. At work, I use my skills and strengths well	High modification index	
Work	30. I am making meaningful contributions at work	Revised: Wording	32. I feel I am making meaningful contributions at work	High modification index	
Work	31. I am learning, improving, and growing at work	Revised: Simplified	33. Through my work, I am growing as a person	High modification index	
Work	32. To the extent my job allows me, I engage in periods of deep focus each day	Revised: Simplified	34. At work, I have regular periods of deep focus		15. At work, I have regular periods of deep focus
Work	33. To the extent my job allows, I use my creativity at work	Revised: Simplified	35. I am able to be creative at work	High modification index	
Work	34. I manage my stress levels well at work	Revised: Wording	36. I manage my emotions at work well		16. I manage my emotions at work well
Work	35. I engage in self-care to minimize burnout at work	Revised: Wording	37. I am able to minimize burnout at work	High modification index	
Work	36. Overall, my work contributes to my well-being	Kept the same	38. Overall, my work contributes to my well-being	High modification index	
Life	37. I am making a meaningful contribution in my life	Revised: Simplified	39. I feel my life is meaningful		17. I feel my life is meaningful
Life	38. I have a sense of purpose in life	Revised: Wording	40. I feel a sense of purpose in my life	High inter-item correlation	
Life	39. I have supportive and satisfying relationships in my life	Revised: Wording	41. I have supportive relationships in my life		18. I have supportive relationships in my life
Life	40. I experience love regularly	Revised: Wording	42. I regularly experience loving connections	High inter-item correlation	
Life	41. I am satisfied with my financial situation	Kept the same	43. I am satisfied with my financial situation		19. I am satisfied with my financial situation
Life	42. I feel safe in my home and in my community	Deleted			
Life	43. I feel a sense of belonging at home and in my community	Deleted			
Life	44. I feel appreciation for my life and the people in it	Revised: Simplified	44. I am grateful for my life	High item-mean	
Life	45. I feel gratitude for my life and the people in it	Revised: Wording	45. I feel compassion for the people in my life	High item-mean	
Life	46. I make enough time to recharge my batteries and enjoy life	Revised: Simplified	46. I take enough time to relax	Item-total correlation low	
Life	47. I have a good work/life balance	Revised: Wording	47. I am satisfied with my work/life balance	High modification index	
Life	48. I have enough novel experiences and adventures in my life	Revised: Wording	48. I enjoy adventures in my life		20. I enjoy adventures in my life
Life	49. I am satisfied with my life as a whole	Kept the same	49. I am satisfied with my life as a whole	High modification index	

Once the wording of all items was finalized, the research team discussed whether further face validity efforts were necessary. All team members agreed that no further changes to the content of the items were necessary, and the next phase of validation began.

### Study 2

#### Objectives

The aim of Study 2 was the establishment of convergent validity of the WCI.

#### Participants and Procedure

An online survey was sent to practicing health and well-being coaches to forward to their clients. The survey was also shared with students enrolled in Wellcoaches courses to become a health and well-being coach. A total of 261 participants responded to the survey (161 clients and 100 students). The average age of participants was 50.6 ± 12.5 years old. The majority (n = 210, 78.1%) were female, while 47 (17.5%) identified as male, and 4 (1.5%) as non-binary. About 3 in 4 participants were white (n = 205, 78.8%), followed by black (n = 18, 6.7%), Asian (n = 12, 4.5%), Hispanic (n = 9, 3.3%) and multiple/other (n = 16, 6.1%). The respondents were mostly located in the United States (n = 238, 91.2%) and Canada (n = 12, 4.6%). No significant differences in demographic factors between client and student participants were detected.

#### Measures

As described above, the WCI with revised items was administered. Each item was measured on an 11-point Likert scale, ranging from 0—Never to 10—Always.

#### Statistical Analysis

A three-step approach to evaluating the items was applied. First, measures of central tendencies (item means and standard deviations), indicators of normality (skewness and kurtosis), along with frequency plots were evaluated. The goal of the evaluation was to check for non-normality and items reaching a potential ceiling effect. Non-normality was assumed if items had exceeded skewness values of 3 and kurtosis values of 10.^11^ Items with a mean score of 9 out of 10 were considered problematic for a potential ceiling effect and removal was recommended.

The second step entailed the calculation of inter-item correlation to check for variance overlap between items. Items with a correlation over .8 were flagged for discussion.^12^ In addition, corrected item-total correlations were conducted to evaluate the fit of the item with each intended dimension. Cut-off values for corrected item-total correlations are discussed in the literature. A conservative cut-off of .5 was set for the study to discuss removal of an item.^13^

The last step included the use of factor analytical techniques to evaluate the theoretical structure of the inventory. Because a theoretical structure (dimensions and respective items) had been established for the present questionnaire by the core HWC team, confirmatory factor analysis (CFA) was chosen.^9^ In addition, Exploratory Structural Equation Modelling (ESEM) was calculated as it permits the cross-loadings of items on several factors, as opposed to CFA, which forces cross-loadings to be 0.^14^

Often CFAs are considered too stringent, whereas ESEM applies a less restrictive approach to estimations of model fit. Yet, ESEM and CFA should not be considered an either/or approach in preliminary analysis of items. Rather, “researchers should compare ESEM and CFA measurement models based on the constructs to be considered” (*P*.104).^15^ The calculation of ESEM and CFAs to test factorial structures has been employed by several questionnaire development and validation studies.^16-18^ Hence, ESEM and CFA were also calculated in the present study.

Following established recommendations, individual items with factor loadings below .60 and/or modification indices over 10 from the CFA were considered for potential removal.^19,20^ Finally, the model fit for both CFA and ESEM were assessed using the following criteria: χ2/df ratio (acceptable fit 2-3, good fit <2)^21^, Incremental Fit Index (IFI, acceptable fit>.90, good fit >.95),^22^ Non-normed Fit Index (NNFI or Tucker Lewis Index, acceptable fit >.90, good fit >.95),^23^ Comparative Fit Index (CFI, acceptable fit >.90, good fit >.95),^23^ and Root Mean Square Error of Approximation (RMSEA, acceptable fit .05-.08, good fit 0.00-0.05).^21^ Reliability was examined using Cronbach’s α. All analyses were conducted in JASP 0.18.3 and MPlus 8.2.

#### Results & Discussion

The analysis of measures of central tendencies (see Table 3) revealed that none of the items violated assumptions of normal distribution. The responses between students and client participants yielded only marginal differences in dimension scores (Mean differences = .07 - .41), hence, the responses were analyzed together. The mean response of 2 items exceeded 9 out of 10. Hence, those items (items 44 and 45) were removed from the scale. Next, 3 items (items 27, 40, and 42) shared elevated inter-item correlations (*r* > .80) and were removed. Another 3 items (items 6, 20, and 47) showed low corrected item-total correlations and were removed as well. In total, 8 items were removed at this step.

**Table 3.** Item Descriptives, Item-Total Correlations, and Factor Loadings – Study 2.

Item	Mean ± SD	Skewness	Kurtosis	Item-Total Correlation	Factor Loadings – CFA	Factor Loadings – ESEM
Mental – Item 1	7.13 ± 1.61	−.53	.13	.57	.57	.47
Mental – Item 2	7.69 ± 1.58	−.79	1.27	.61		
Mental – Item 3	7.55 ± 1.96	−.97	.75	.80	.88	.81
Mental – Item 4	8.15 ± 1.56	−1.06	1.25	.80		
Mental – Item 5	7.71 ± 1.89	−1.31	2.27	.68	.71	.57
Mental – Item 6	8.40 ± 1.37	−1.28	2.82	.45		
Mental – Item 7	7.42 ± 1.69	−.77	.75	.69	.71	.77
Mental – Item 8	7.99 ± 1.51	−1.06	1.99	.50		
Mental – Item 9	6.97 ± 2.10	−.77	.10	.75	.81	.86
Mental – Item 10	8.10 ± 1.56	−1.25	2.09	.67		
Mental – Item 11	6.93 ± 2.11	−.77	−.13	.55		
Mental – Item 12	8.56 ± 1.41	−1.26	1.95	.62		
Mental – Item 13	8.54 ± 1.56	−1.52	2.62	.69		
Mental – Item 14	8.11 ± 1.65	−1.60	3.17	.79		
Physical – Item 15	7.29 ± 1.85	−.92	.88	.68		
Physical – Item 16	7.39 ± 2.29	−.93	.17	.66	.75	.79
Physical – Item 17	7.63 ± 2.19	−.89	.13	.52	.63	.65
Physical – Item 18	7.02 ± 2.62	−.87	.07	.62	.64	.59
Physical – Item 19	7.02 ± 2.24	−.91	.54	.64		
Physical – Item 20	7.64 ± 2.19	−1.21	1.30	.31		
Physical – Item 21	7.48 ± 2.23	−1.14	.97	.56	.67	.57
Physical – Item 22	7.71 ± 2.02	−1.13	1.16	.81		
Work – Item 23	7.65 ± 1.88	−1.40	2.70	.71		
Work – Item 24	7.87 ± 1.94	−1.53	3.05	.82	.88	.78
Work – Item 25	8.09 ± 1.94	−1.71	3.45	.70	.67	.83
Work – Item 26	8.10 ± 1.75	−1.80	4.79	.85	.90	.92
Work – Item 27	8.08 ± 1.92	−1.53	2.60	.88		
Work – Item 28	7.94 ± 1.84	−1.69	3.33	.76	.78	.70
Work – Item 29	7.69 ± 2.02	−1.47	2.47	.77	.72	.77
Work – Item 30	8.25 ± 1.72	−1.66	3.74	.73		
Work – Item 31	8.32 ± 1.66	−1.99	5.94	.80		
Work – Item 32	8.26 ± 1.82	−1.96	4.85	.77		
Work – Item 33	8.11 ± 2.10	−1.77	3.18	.80		
Work – Item 34	7.58 ± 2.04	−1.33	2.06	.70	.68	.70
Work – Item 35	7.52 ± 2.29	−1.33	1.65	.73		
Work – Item 36	8.13 ± 1.78	−1.78	4.21	.67	.70	.55
Work – Item 37	7.09 ± 2.25	−1.13	.89	.77		
Work – Item 38	7.24 ± 2.31	−1.11	.91	.65		
Work – Item 39	7.48 ± 2.30	−1.27	1.14	.87		
Life – Item 40	8.30 ± 1.73	−1.25	1.63	.79	.88	.67
Life – Item 41	8.26 ± 1.78	−1.20	1.13	.77		
Life – Item 42	8.53 ± 1.72	−1.69	3.29	.76	.78	.97
Life – Item 43	8.15 ± 1.98	−1.30	1.42	.74		
Life – Item 44	7.21 ± 2.40	−1.10	.79	.55	.53	.58
Life – Item 45	9.03 ± 1.42	−2.21	6.31	.71		
Life – Item 46	9.06 ± 1.05	−1.31	1.98	.59	.57	.40
Life – Item 47	7.29 ± 2.24	−.98	.49	.49		
Life – Item 48	8.04 ± 1.95	−1.25	1.27	.61		
Life – Item 49	8.21 ± 1.81	−1.44	2.10	.86		

Next, a CFA was conducted with the remaining 41 items. The overall fit of the model was unsatisfactory (χ^2^/df ratio = 8.82, IFI = .81, NNFI = .80, CFI = .81, RMSEA = .09 95% CI = .08 - .09). Hence, the factor loadings and modification indices of each item were evaluated. This led to the removal of 2 further items (items 10 and 15) due to low factor loadings and 19 items (items 2, 4, 8, 11, 12, 13, 14, 19, 22, 23, 28, 29, 30, 31, 32, 33, 35, 37, and 38) due to high modification indices. In sum, 21 items were removed in this step.

While the reduction stage is intended to reduce the number of items, it should be noted that the removal of items based on modification indices is discussed in the literature and should only be done with theoretical justification.^13^ As such, each item was evaluated carefully with an eye on content overlap to the items that were retained. In particular, the research team examined the semantic and theoretical closeness of 2 items before removal. Additionally, we calculated the correlation between the dimension scores with all 49 items and the scale without the removed items, following recommendations by Smith and colleagues.^24^ The correlations between the dimensions exceeded .92, indicating that at least 83% of the variance of subscales was retained after item removal. The theoretical considerations and the evidence from the correlation provide support for the decision to remove the items due to high modification indices.

The remaining 20 items were subjected to another CFA and ESEM. The model showed a good fit of the data (χ^2^/df ratio = 1.21, IFI = .94, NNFI = .91, CFI = .94, RMSEA = .06 95% CI = .05 - .07). All items loaded well on their respective dimensions (>.57). The correlations between the dimensions ranged from .33 to .64, indicating sufficient uniqueness (shared variance between dimensions <42%) of each dimension. The exploratory structural equation model showed an excellent model fit of the data (χ^2^/df ratio = 1.54, NNFI = .96, CFI = .98, RMSEA = .05 95% CI = .03 - .06). Cross-loadings of items were minimal (<.2). The Cronbach’s α for each dimension ranged from .77 to .90, indicating satisfactory internal consistency.

The objective of the second study was to provide evidence for convergent validity, by selecting items that best reflect the unobservable, perceived construct. Reducing items should be done with caution, balancing brevity and time efficiency of the potential administration of the inventory with theoretical depth of content. In essence, it should be brief enough for time-efficient administration yet cover the fundamental aspects of the theoretical underpinnings of the phenomenon under investigation.

By reducing the number of items from 49 to 20, the scale is much shorter and will be easier to use, however, through theoretical discussions and statistical evidence face validity was preserved. In addition, the results from the CFA and ESEM showed satisfactory evidence for the factorial structure of the 20-item version of the scale, supporting its convergent validity. As such, we proceeded to the final phase of the validation process.

### Study 3

#### Objectives

Confirming the convergent validity of the 20-item version of the WCI and testing the predictive validity of the scale.

#### Participants and Procedure

An online survey was shared broadly to include subscribers to a monthly Wellcoaches newsletter and on social media. The aim was to reach participants between 25 and 65 years of age and to capture a large sample of the full-time working population. A total of 531 participants responded to the survey. The average age of the respondents was 49.8 ± 10.6 years old. The majority (n = 473, 89.1%) were female, while 52 (9.8%) identified as male, and 4 (.8%) as non-binary. Over 80% of the participants were white (n = 430, 81.0%), followed by black (n = 19, 3.6%), Asian (n = 28, 5.3%), Hispanic (n = 18, 3.4%) and multiple/other (n = 32, 6%). The respondents were mostly located in the United States (n = 484, 91.1%) and Canada (n = 12, 2.3%). Most of the participants completed a Bachelor’s (n = 181, 34.2%), Master’s (n = 258, 48.6%), or Doctoral (n = 65, 12.2%) degree, while 25 participants held a high-school degree (4.7%). About 2 out of 3 participants were working full-time (n = 340), while 102 (19.4%) worked part-time and 18 (3.4%) were unemployed.

#### Measures: Well-being

The 20-item version of the WCI was utilized. As outlined in Table 4, the scale consists of 4 subdimensions: Mind (5 items), Body (4 items), Work (7 items), and Life (4 items). As in Study 2, the items were assessed on an 11-point Likert scale, ranging from 0 – Never to 10 – Always.

**Table 4.** Item Descriptives, Item-Total Correlations, and Factor Loadings – Study 3.

Item	Mean ± SD	Skewness	Kurtosis	Factor Loadings – CFA	Factor Loadings – ESEM
Mental – Item 1	6.95 ± 1.54	−.58	1.17	.61	.43
Mental – Item 3	7.29 ± 1.83	−.84	.52	.77	.91
Mental – Item 5	7.83 ± 1.52	−.83	1.08	.66	.56
Mental – Item 7	7.25 ± 1.56	−.77	.83	.67	.51
Mental – Item 9	6.86 ± 1.87	−.87	.99	.75	.79
Physical – Item 16	7.50 ± 2.21	−.91	.23	.80	.78
Physical – Item 17	7.64 ± 2.18	−.88	.27	.73	.71
Physical – Item 18	6.96 ± 2.65	−.76	−.27	.72	.75
Physical – Item 21	7.48 ± 2.13	−.90	.52	.60	.48
Work – Item 24	7.90 ± 1.84	−1.26	1.94	.82	.81
Work – Item 25	7.98 ± 1.93	−1.51	2.76	.74	.76
Work – Item 26	8.00 ± 1.70	−1.41	2.75	.89	.93
Work – Item 28	7.93 ± 1.68	−1.53	3.08	.73	.67
Work – Item 29	7.70 ± 1.98	−1.25	1.80	.78	.79
Work – Item 34	7.37 ± 2.03	−1.10	1.41	.70	.65
Work – Item 36	8.22 ± 1.61	−1.30	2.27	.59	.41
Life – Item 40	8.20 ± 1.61	−1.29	2.27	.82	.63
Life – Item 42	8.44 ± 1.68	−1.48	2.31	.72	.82
Life – Item 44	8.90 ± 1.17	−1.38	2.77	.64	.65
Life – Item 46	7.99 ± 1.97	−1.18	1.21	.52	.44

#### Life satisfaction

Life satisfaction was measured with the Satisfaction with Life Scale (SWLS) by Diener et al.^25^ This five-item scale (example item: In most ways my life is close to my ideal), is measured on a 7-point Likert Scale ranging from 1—Strongly disagree to 7—Strongly agree. The SWLS is a widely used instrument with evidence of good reliability and validity.^26^ In the present study, an excellent internal consistency (Cronbach’s α = .91) was detected.

#### Depression

The PHQ-8^27^ is an 8-item scale which measures the prevalence of depressive symptoms over the past 2 weeks. It derived from a nine-item scale (PHQ-9), however, the removal of 1 item for brevity does not affect its clinical sensitivity and specificity.^28^ On each item, the participants rate the frequency of the symptom, ranging from 0—Not at all to 3—Nearly every day (example item: Little interest or pleasure in doing things). The total score is the sum of all items. The PHQ-8 has demonstrated excellent reliability and validity.^29^ In the present study, a satisfactory internal consistency (Cronbach’s alpha = .84) was detected.

#### Perceived Stress

Stress was measured with the Perceived Stress Measure (PSM-9),^30^ a 9-item measure. The participants rated their symptoms of stress over the past 4 to 5 days. Each item (example item: I feel stressed) is measured on an 8-point Likert scale ranging from 1—Not at all to 8—Extremely. Evidence supports the reliability and validity of the PSM-9 as a measure of stress.^31^ Accordingly, the PSM-9 showed satisfactory internal consistency (Cronbach’s α = .88) in the present study.

#### Statistical Analysis

To provide further support for the convergent validity and factorial structure of the 20-item version of the inventory from Study 2, the data was subjected to CFA and ESEM. The same goodness-of-fit indices as in Study 2 were used.

To provide evidence of predictive validity, measures of life satisfaction, depression, and stress were collected from the participants. Conceptually, life satisfaction and well-being should be positively related,^32^ while depression^33^ and perceived stress^34^ should be negatively associated with well-being. Pearson correlation coefficients were calculated to test the relationships between the subdimensions of the WCI and life satisfaction, depression, and perceived stress.

#### Results & Discussion

All items were sufficiently distributed for parametric analyses (Kurtosis <4, Skewness <2). All item descriptives are summarized in Table 4. The model showed a satisfactory fit of the data (χ^2^/df ratio = 3.29, IFI = .92, NNFI = .91, CFI = .92, RMSEA = .07 95% CI = .06 - .07). All items loaded well on their respective dimensions (>.52). The correlations between the dimensions ranged from .43 to .61, indicating sufficient uniqueness (shared variance between dimensions <38%) of each dimension. The structural equation model showed a good model fit of the data (χ^2^/df ratio = 2.81, NNFI = .93, CFI = .96, RMSEA = .06 95% CI = .05 - .07).

All goodness-of-fit indices (from Study 2 and 3) are summarized in Table 5. Cross-loadings of items were minimal (<.22). The Cronbach’s alphas for each dimension ranged from .76 to .90, indicating satisfactory internal consistency. Overall, the findings support the convergent validity of the 20-item version of the WCI in the present sample.

**Table 5.** Goodness of Fit Indices for Study 2 and Study 3.

	χ2/df	NNFI	CFI	RMSEA	95% CI
Study 2 – CFA (41 Items)	8.82	.80	.81	.09	.08-.09
Study 2 – CFA (20 Items)	1.21	.91	.94	.06	.05-.07
Study 2 – ESEM (20 Items)	1.54	.96	.98	.05	.03-.06
Study 3 – CFA (20 Items)	3.29	.91	.92	.06	.05-.07
Study 3 – ESEM (20 Items)	2.81	.93	.96	.06	.05-.07

The correlation analysis revealed the theoretically anticipated relationships (see Table 6). The life satisfaction measure shared significant positive relationships (r = .43 - .69) with the dimensions of the WCI. Perceived stress (r = −.43 - .56) and depression (r = −.45 - .47) shared significant negative relationships with the dimensions of the WCI. The findings support the predictive validity of the scale, as anticipated relationships based on theory could be confirmed statistically.

**Table 6.** Correlations Between Constructs.

Variable	1	2	3	4	5	6
1. WCI - Mental Well-Being						
2. WCI - Physical Well-Being	.43					
3. WCI - Work Well-Being	.61	.40				
4. WCI - Life Well-Being	.55	.44	.56			
5. Life Satisfaction	.51	.43	.52	.69		
6. Depression	−.47	−.47	−.45	−.45	−.51	
7. Stress	−.56	−.43	−.50	−.46	−.52	.67

Note: All correlations *P* < .001.

## General Discussion

The purpose of the 3 outlined studies was to provide psychometric testing and validation of the WCI, a proposed measure of whole-person well-being in the context of HWC. The first study outlined procedures for creating a measure with items that are readable, applicable, and understandable while reflecting the essence of well-being. This study’s results support the face validity of the WCI.

The second study included the statistical evaluation of the items and an examination of the fit to each dimension. This step resulted in a reduction of items from 49 to 20. The data showed evidence supporting convergent validity of the shortened scale. Using a large sample (N > 500), the last study provided more evidence for convergent validity (i.e., through the confirmation of the factorial structure of the previous step) and further supported the predictive validity of the scale, as anticipated correlations to other concepts (i.e., depression, stress, and life satisfaction) were found. Therefore, the WCI is a valid, applicable, and reliable scale for use in HWC research and practice.

A large heterogeneity in HWC outcome measures may be attributable to a lack of measures specifically designed for HWC.^35^ Researchers in HWC are generally concerned with using valid and reliable measures of central outcomes of HWC interventions. Given the results of our study, it is recommended that researchers broadly adopt the 20-item version of the WCI as a key outcome measure in future HWC trials. The WCI serves as the first specifically designed HWC core outcome measure. Large data collection on the WCI would enable comparison of HWC effect sizes among different populations (e.g., obesity, cancer) and trial designs (e.g., length, frequency). In addition, meta-analytic evidence of HWC would benefit from less heterogeneity in outcome measures, as a core measure improves the comparability between studies. As such, the WCI will help further our understanding of the benefits of HWC.

From a practice perspective, health and well-being coaches are advised to consider using the 20-item version WCI or the longer 49-item version depending on their purpose. As a measurement tool, the 20-item version is recommended as a reliable and valid instrument that enables the comparison between subgroups and/or over time. For example, to simply determine the effectiveness of HWC services use the shorter WCI. In addition, if practitioners seek to compare measures among or between patients, the shortened version is again best used. However, the 49-item version of the WCI can be used as a powerful coaching tool. When applied as part of a patient or client’s intake process and later as part of their evaluation process, the longer WCI may be useful and preferred over the shorter version.

**Figure 2.**
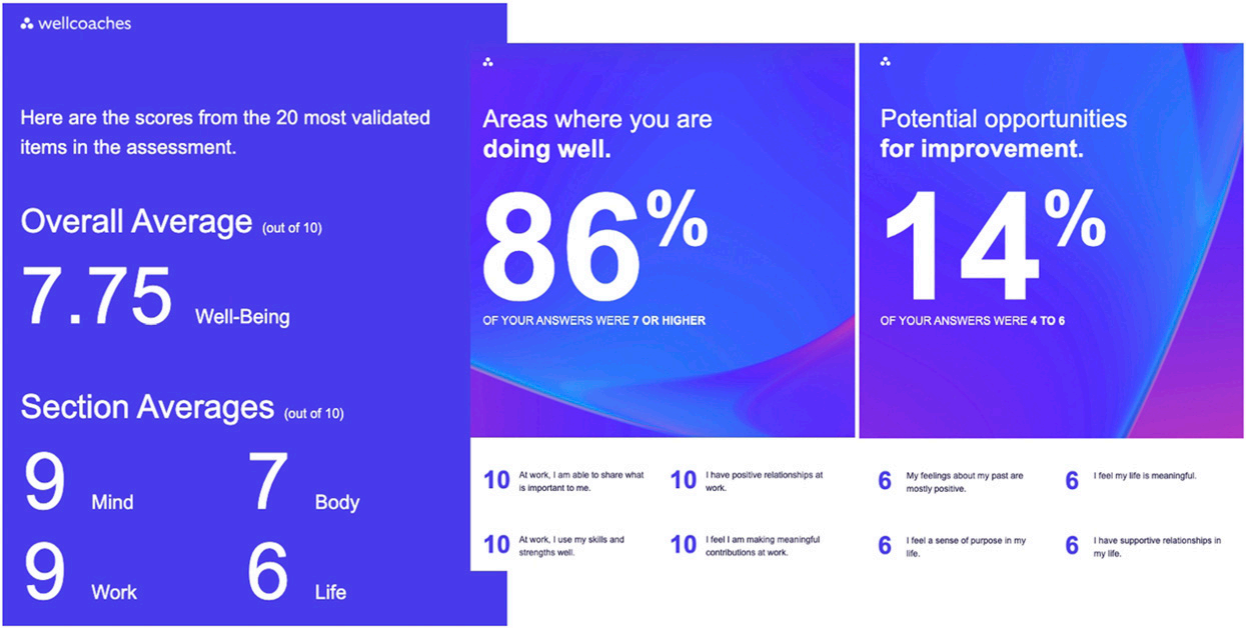
WCI sample results summary.

With a greater number of HWC relevant constructs in the 49-item version, the coach may find a topic that is salient to their client’s path to better well-being using the longer WCI. Coaches should consider the longer version of the WCI if they have the time and prefer greater depth of information for and from their clients. If the 49-item WCI is completed, only the scoring of the 20-item version of the scale is recommended for longitudinal tracking, practical comparison, and research purposes. Therefore, the WCI can be applied to an individual thinking about their own well-being, or as a population evaluation of well-being. The WCI is openly available online https://survey.alchemer.com/s3/7971712/Wellcoaches-Well-being-Inventory. Results are emailed to everyone completing the inventory, including average overall score, based on the core 20 items and average score in each subdimension. Results also include suggestions for where the individual is doing well, needs improvement, or may be near burnout (see Figure 2). For research purposes, the collection of large datasets for normative data would deliver benchmark scores for individual and collective comparisons. Such research could also inform the design of future interventional studies which may employ the WCI, as it provides comparative values for the assessment of well-being levels in potential participants.

The results of the present series of studies should be interpreted considering several limitations. First, the WCI was developed by a team of researchers who bring extensive experience in HWC and questionnaire development. However, the team also lacked diversity, which is a limitation as bias in the creation of the items might have been present. The series of studies recruited more than 80% of its participants from a non-client population to ensure feasibility. Further validation efforts should test the validity and reliability of the WCI in a larger client population. In addition, the samples from all studies were predominantly working age, female, educated, and white. While we consider the WCI to be generalizable, additional testing in males, older (e.g., above 65) and younger (e.g., university students) people, diverse and minoritized groups, and less educated individuals may be desirable. In addition, it may be desirable to test the reliability and validity of the scale in countries outside of North America. In this context, it may be important to adapt the WCI-Work subscale to be relevant to school-age education or retirement activities. In addition, the longitudinal stability of the WCI should also be examined. Furthermore, future research should explore the relationship to other related constructs (e.g., thriving), which was beyond the scope of the present study. Finally, the WCI query of love, hope, meaning, gratitude, and compassion is considered to embody an expression of spirituality. However, spirituality, as related to religious practices or an independent concept, was not questioned in the WCI. This is a limitation given the important potential implications of religious practices for some groups, cultures, and individuals.

In summary, the WCI is a valid, applicable, and reliable tool to measure well-being as a core HWC outcome variable. The WCI is also a coaching tool allowing clients to identify relevant categories/items addressable during the coaching process to encourage individual thriving and flourishing. The inventory is available free of charge and can be found in the appendix to this study. Researchers are encouraged to use the WCI in all HWC studies while practitioners should use the WCI widely as a coaching tool and outcome measure.

## Supplemental Material

Supplemental Material - The Well-Being Coaching Inventory (WCI): Questionnaire Development and Validation Supplemental Material for The Well-Being Coaching Inventory (WCI): Questionnaire Development and Validation by Sebastian Harenberg, Gary Sforzo, Rosie Hunter, Erika Jackson, and Margaret Moore in American Journal of Lifestyle Medicine
