# Supplementary material for: The Well-Being Coaching Inventory (WCI): Questionnaire Development and Validation
Source: Am J Lifestyle Med. 2025 Feb 20;19(6):908–25. doi: 10.1177/15598276251320573 (PMC11843568; doi:10.1177/15598276251320573)
Supplement: Supplemental Material - The Well-Being Coaching Inventory (WCI): Questionnaire Development and Validation [file sj-pdf-1-ajl-10.1177_15598276251320573.pdf]

Appendix

The following questions measure your well-being in four dimensions: mental, physical, work, and life well-being. Please respond to each statement as it best describes your well-being over the past 3 months. Rate each statement from 0 (Never) to 10 (Always). Simply be honest and accurate as possible - there are no right or wrong answers.

1. My mind is present throughout the day. (Mental)
2. I accept myself as I am, with my strengths and limitations (Mental)
3. I view setbacks as learning opportunities. (Mental)
4. I can calm myself down when I feel stressed or anxious. (Mental)
5. I am kind to myself in difficult times. (Mental)
6. I am physically active most days. (Physical)
7. I eat several servings of vegetables/fruits daily. (Physical)
8. I feel I maintain a healthy weight. (Physical)
9. I spend time in nature regularly. (Physical)
10. I feel inspired to be my best at work. (Work)
11. I have enough freedom to choose how I do my job. (Work)
12. I find ways to make my work fulfilling. (Work)
13. I am satisfied with my work performance. (Work)
14. At work, I am able to share what is important to me. (Work)
15. At work, I have regular periods of deep focus. (Work)
16. I manage my emotions at work well. (Work)
17. I feel my life is meaningful. (Life)
18. I have supportive relationships in my life. (Life)
19. I am satisfied with my financial situation. (Life)
20. I enjoy adventures in my life. (Life)
